# Effects of a Multimodal Exercise Intervention on Single- and Dual-Task Gait Performance in Older Patients with Open-Angle Glaucoma: A Pilot Study

**DOI:** 10.3390/geriatrics11040095

**Published:** 2026-07-30

**Authors:** Constantin W. Freitag, Martin Behrens, Robert Bielitzki, Tom Behrendt, Khaldoon O. Al-Nosairy, Cynthia Moffack Djuloun, Francie H. Stolle, Hagen Thieme, Michael B. Hoffmann, Lutz Schega

**Affiliations:** 1 Department of Sport Science, Institute III, Otto von Guericke University Magdeburg, Zschokkestraße 32, 39104 Magdeburg, Germany; constantin.freitag@ovgu.de (C.W.F.); tom.behrendt@ovgu.de (T.B.); 2 Division for Research Methods and Analysis Procedures in Sports Science, University of Applied Sciences for Sport and Management Potsdam, Olympischer Weg 7, 14471 Potsdam, Germany; behrens@fhsmp.de; 3 Department of Orthopaedics, University Medical Centre, Doberaner Straße 142, 18057 Rostock, Germany; 4 Department of Human Movement Science and Exercise Physiology, University of Hamburg, Turmweg 2, 20148 Hamburg, Germany; robert.bielitzki@uni-hamburg.de; 5 Section for Clinical and Experimental Sensory Physiology, Ophthalmic Department, University Hospital Magdeburg, Leipziger Str. 44, 39120 Magdeburg, Germany; khaldoon.al-nosairy@med.ovgu.de (K.O.A.-N.); cynthia.moffack@st.ovgu.de (C.M.D.); francie.stolle@med.ovgu.de (F.H.S.); hagen.thieme@med.ovgu.de (H.T.); michael.hoffmann@med.ovgu.de (M.B.H.); 6 Center for Behavioral Brain Research, Universitätsplatz 2, 39106 Magdeburg, Germany

**Keywords:** cognition, resistance training, motor-cognitive training, overground walking

## Abstract

**Background/Objectives**: To investigate the effects of a 12-week multimodal intervention (MMI [resistance + motor-cognitive dual-task training]) versus a unimodal intervention (UMI [resistance training]) on gait and cognitive performance in people with glaucoma during single-task (ST) and dual-task (DT) walking. **Methods**: In this randomized controlled pilot study, fifteen glaucoma patients (MMI: *n* = 8, UMI: *n* = 7) completed 24 supervised intervention sessions over 12 weeks. Spatio-temporal gait parameters (stride length, gait velocity, minimum toe clearance [MTC], and their respective coefficient of variance [CoV]) were assessed using inertial measurement units (sampling frequency 100 Hz) during ST and DT walking. During DT walking, the participants performed three cognitive tasks: reaction time task, N-Back task, and letter fluency task. Each cognitive task was performed with two levels of difficulty. Repeated measures analysis of covariance (TIME × INTERVENTION × CONDITION) was conducted to analyze the data. **Results**: No significant group or time effects were observed for ST walking or cognitive performance. Independent of intervention, dual-task costs (DTC) improved for MTC (*p* = 0.188, $\eta_{p}^{2}$ = 0.205) and MTC_CoV_ (*p* = 0.021, $\eta_{p}^{2}$ = 0.713) over time. UMI showed greater improvements over time than MMI for DTC MTC (*p* = 0.168, $\eta_{p}^{2}$ = 0.223) and DTC MTC_CoV_ (*p* = 0.047, $\eta_{p}^{2}$ = 0.407). **Conclusions**: This pilot study indicated that both MMI and UMI decreased DTC MTC and DTC MTCCoV in glaucoma patients, with probably higher effects after UMI. These preliminary findings must be interpreted with caution due to the small sample size, but the provided effect size measures and sample size calculations can be used for future randomized controlled trials to verify and extend these results.

## 1. Introduction

Glaucoma comprises neurodegenerative eye diseases that are characterized by a thinning of the retinal ganglion cell layer ultimately resulting in visual field loss [1,2]. It is considered the leading cause of irreversible blindness worldwide [3] and expected to affect 111.8 million people by 2040 [4].

Glaucomatous visual field loss is characterized by insidious onset [5] with progressive deterioration [4,6,7]. Primary risk factors include age and elevated intraocular pressure (IOP) [4,8]. Increase in IOP results from elevated outflow resistance, primarily within the trabecular meshwork pathways. This increase in pressure can lead to deformation of the lamina cribrosa, and thus, to the degeneration of retinal ganglion cells and subsequent visual field damage [2].

Since visual input is essential for human locomotion, the glaucoma-related degeneration of the retinal ganglion cells may impair mobility, including gait performance and increase the risk of falls as the disease progresses [9,10,11]. Further, growing evidence supports a strong morpho-functional connection between eye and brain in glaucoma disease, indicating shared neurodegenerative mechanisms that may contribute to cognitive impairments similar to those seen in Alzheimer disease [12]. Given that cognition also plays a critical role in locomotion, cognitive deficits may further impair postural control and increase the risk of falls [13,14].

Several spatio-temporal gait parameters, including stride length, gait velocity, and gait variability measures have been identified as predictors for falls [15,16]. Among these, particularly the minimum toe clearance (MTC) has emerged as a promising indicator for the evaluation of motor control during walking [17] and potentially predicting the risk of falling in glaucoma patients [18]. MTC refers to the smallest vertical distance between the toe and ground during the mid-swing phase of the gait cycle [15]. A lower MTC and greater MTC variability are both associated with an increased likelihood of falling [17]. Several studies have already examined the effects of glaucoma on gait performance [9,19,20,21,22]. For example, glaucoma severity, quantified using visual field sensitivity, has been associated with a wider base of support and increased gait variability [22]. Notably, to the knowledge of the authors, only Gomes et al. [20], Lee et al. [23], and Freitag et al. [18] have investigated the effect of glaucoma on gait performance compared to healthy controls. While Gomes et al. [20] (i.e., 5.74 m walking at comfort velocity for six trials) and Freitag et al. [18] (i.e., walking at comfort velocity back and forth over a 10 m track for 3 min) found no group differences in gait velocity and step length, Lee et al. [23] (i.e., 5 m walking at comfort velocity) showed an impaired walking performance in glaucoma patients (e.g., slower gait velocity). However, only Freitag et al. [18] assessed gait performance during single-task (ST) as well as motor-cognitive dual-task (DT) walking and evaluated the MTC, revealing an increased MTC variability in glaucoma patients. Interestingly, Freitag et al. reported those effects in glaucoma patients with only small visual defects.

Gait performance has frequently been assessed during DT walking [24,25,26], which involves performing a cognitive task and walking. This often leads to an increased gait variability [15,27]. The decline in gait performance under such conditions may be attributed to reduced cognitive resources available for the motor task, which can be explained by the central capacity sharing model and/or the bottleneck model describing the parallel and/or sequential neural processing of the motor and cognitive interference task [28]. As a result, performance typically deteriorates at least in one of the tasks, a phenomenon known as dual-task costs (DTC). This is of particular relevance in activities of daily life, where DT situations such as talking on the phone while walking are common. Notably, a low DT walking performance, i.e., higher DTC, is associated with a higher risk of falling [29].

Taken together, with progressive disease development, glaucoma patients appear to have an increased risk of falling due to glaucomatous visual field defects as well as potential cognitive impairments [18]. In this regard, resistance training appears to be a promising intervention since it has been shown to improve postural control and gait performance in older adults [30] and to exert beneficial effects on cognitive performance [31]. For DT training (i.e., the simultaneous execution of motor and cognitive tasks) there is evidence that it elicits structural and functional changes in the aging brain, which were associated with an improved cognitive performance [32,33,34,35]. Although the evidence is inconclusive, DT training might be a more promising approach for enhancing cognitive performance than single motor or cognitive training [36,37].

A recently published systematic review highlighted the effects of combining resistance and cognitive training, distinguishing between two approaches: (i) simultaneous intervention (resistance and cognitive training on the same day) and (ii) sequential interventions (resistance and cognitive training on separate days) [38]. The authors concluded that simultaneous training at moderate to high intensity, for 2–3 times per week for at least 30 min appears to be the most effective regarding strength, gait, balance processing speed, attention and executive function in older adults [38]. However, most studies investigated ST cognitive training, while only three examined DT cognitive training [38]. Moreover, these three studies used only passive control groups [39,40,41], which limit the interpretation of the results, and have not included glaucoma patients.

In conclusion, a multimodal intervention (MMI), i.e., the combination of resistance and motor-cognitive DT training [42], might be more effective than a unimodal intervention (UMI), i.e., resistance training alone, in improving gait performance, here referred as the primary outcome, and cognitive performance (secondary outcome) with the ultimate goal of reducing the risk of falls.

The aim of the present study was to investigate the effect of MMI and UMI on ST and DT walking performance as well as cognitive performance in glaucoma patients. Based on the evidence regarding the effects of DT training [42] and resistance training [31], it was hypothesized that in glaucoma patients MMI leads to higher improvements in gait performance, especially during DT walking, and in cognitive performance than UMI.

## 2. Materials and Methods

### 2.1. Trial Design

This two-arm randomized controlled pilot trial was conducted between August 2020 and December 2022. All interventions and assessments took place at the Department of Sport Science, Otto von Guericke University Magdeburg, and were carried out in accordance with the Declaration of Helsinki. Ethical approval was granted by the Ethics Committee of the University Medical Faculty Magdeburg (32/18). The study is reported following the CONSORT guidelines for randomized pilot trials [43]. The data presented here is part of a broader investigation examining the effects of MMI versus UMI on visual, motor, and cognitive performance, as well as on structural and functional brain adaptations in glaucoma patients and healthy controls (German Clinical Trial Register, ID: DRKS00022519, registered 5 August 2020, https://drks.de/search/de/trial/DRKS00022519, accessed on accessed on 7 May 2026). Owing to the small sample size and the complexity of statistical analyses, results for the healthy control group were reported separately [44].

### 2.2. Participants

**Figure 1:**
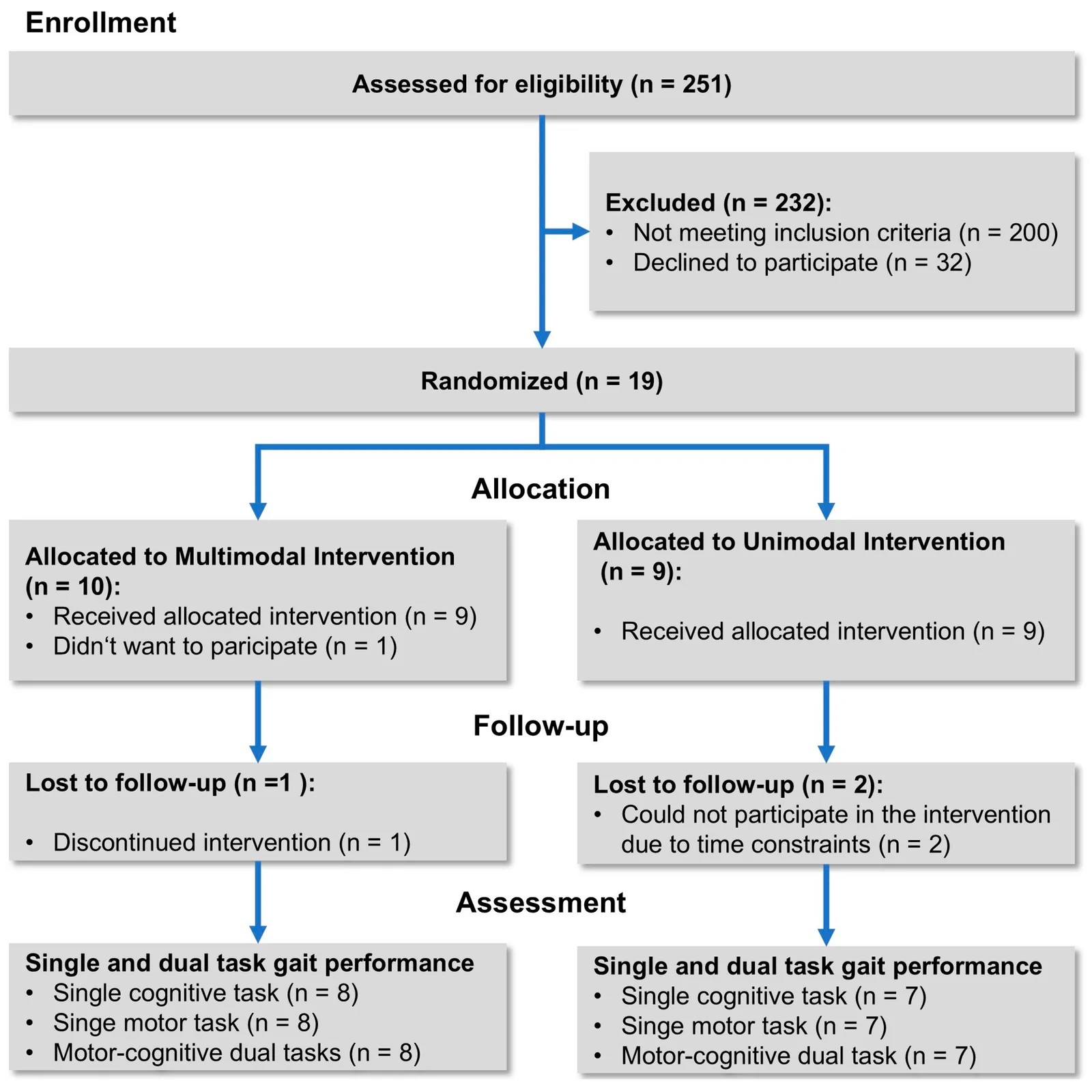
Study schedule of enrollment, intervention, and assessments.

The study schedule for enrollment, intervention, and assessments followed the structure outlined in Figure 1. The sample size was not derived from a dedicated analysis, but was informed by the results of a prior study conducted by Demiracka and colleagues [45], which involved a group comparison of 11 control participants and 21 individuals undergoing Life Kinetik^®^ training. That study reported significant intervention-related changes in brain connectivity. It is important to note that in the present study the period of data collection and funding overlapped with the COVID-19 pandemic, which limited the achievable sample size to a maximum of 19 participants. Therefore, the outcomes of the randomized controlled trial will be treated and reported as a pilot study.

Participants were recruited at the Department of Ophthalmology at the University Hospital Magdeburg, local ophthalmologists, and the national patient network. Ophthalmic workup included slit-lamp eye examination of anterior and posterior segments as well as assessments of best corrected visual acuity (BCVA), visual field (VF, Humphrey visual field analyzer^®^, Jena, Germany), and peripapillary retinal nerve fiber layer thickness [pRNFL, Spectralis^®^ optical coherence tomography (OCT), Heidelberg, Germany]. Inclusion criteria for participants were: (i) age ≥ 60 years, (ii) diagnosis of open angle glaucoma, (iii) visual field defects (see Table 1. for ophthalmological characteristics), and (iv) the ability to walk at least 6 min without support. Participants were excluded based on the following criteria: (i) eye trauma or surgery (but not cataract and glaucoma surgery), (ii) other eye diseases (but not incipient cataract), (iii) neurological disorders, (iv) stroke, (v) cardiovascular diseases, (vi) rheumatism, as well as (vii) orthopedic diseases (e.g., arthrosis (grade II or higher), musculoskeletal impairment, tendinitis, tenosynovitis, myositis, prosthesis and joint replacements in the lower extremities). The allocation was performed at the Department of Sport Science at the University of Magdeburg. All participants signed the informed consent form before participating in this study and were randomly assigned to either the MMI or the UMI using counterbalanced randomization (allocation ratio was 1:1) by a computer-generated table of random numbers, see Figure 1.

### 2.3. General Procedure

Data were recorded prior to and following (within 3 weeks, respectively) the 12-week intervention period. Each measurement started with participants filling the Freiburger Questionnaire on Physical Activity [46] to assess the level of daily physical activity. Moreover, anthropometric data of the participants were recorded. The detailed testing procedure with the methods for obtaining spatio-temporal gait parameters has been fully described by Freitag et al. [18]. However, in brief, over two consecutive days, participants performed ST walking and DT walking at a comfortable velocity over a 10 m track back and forth for 3 min, respectively. During DT walking, the participants performed three cognitive tasks (outcomes): (i) reaction time task (s), (ii) N-Back task (correct responses), and (iii) letter fluency task (correct responses) while walking the track. Each cognitive task was performed with two levels of difficulty.

The reaction time task required participants to respond to target tones, with difficulty manipulated via inter-stimulus intervals (3 s vs. 2 s). Working memory [47,48] was assessed using modified 1-back (easy) and 2-back (severe) tasks, in which participants responded to repeated number sequences. Only correct responses were analyzed. To produce a response, participants had a computer mouse in each hand. A modified letter fluency task assessed executive functions, including inhibition, working memory, and cognitive flexibility [49]. Participants generated as many words as possible beginning with specified letters. Difficulty was increased by requiring alternating responses between two letters. Responses were audio-recorded and analyzed according to predefined linguistic criteria.

In order to calculate the DTC for participants’ cognitive performance, cognitive tasks were also completed while sitting (i.e., cognitive ST). Thus, each cognitive task was performed under ST and DT conditions and two levels of difficulty (easy followed by severe), yielding four trials per task. During all walking conditions, spatio-temporal gait parameters (i.e., stride length, gait velocity, MTC), and their respective coefficient of variation (CoV) were assessed using inertial measurement units (XSENS MTW Awinda, Movella, Delft, The Netherlands).

Due to the COVID-19 pandemic, participants were advised to wear a FFP2 face mask. The assessors of the measurements were not blinded to group allocation because they also supervised the interventions.

### 2.4. Intervention

Participants in both intervention groups underwent a 12-week program with two sessions per week (i.e., a total of 24 sessions) on non-consecutive days. Each exercise session lasted 60 min and was supervised by experienced instructors. All details regarding the intervention can be found in Freitag et al. [44]. Briefly, the MMI intervention contained DT training based on the Life Kinetik^®^ program [50] and resistance training. The training sessions included exercises such as the following: after an auditory cue (e.g., “1,” “tree,” “3,” “Munich”), participants were required to move a specific limb (e.g., right arm = 1, left arm = tree, left leg = 3, right leg = Munich). If at least 6 out of 10 trials were performed correctly, the task was progressed to a more advanced level. The UMI consisted of resistance training only. The resistance training was metronome-paced at 30 bpm (7 repetitions, 2 s eccentric/2 s concentric, 2 sets per exercise) using free weights and exercise machines in a fixed order. Because exercising in a supine position affects the IOP, all exercises were performed while sitting [51,52]. Nine resistance exercises were selected to target all major muscle groups [53] (Supplementary Table S1). Exercise intensity was prescribed using a rating of perceived exertion (RPE) CR-10 Borg scale [54] and should be moderate to somewhat severe (3–4). During MMI, to increase the DT training intensity, the duration of the DT training increased and the resistance training decreased from month to month, see Supplementary Table S2.

The UMI included a standardized 10 min warmup, consisting of walking at a fast pace and dynamic stretching of the lower and upper extremities. A cool-down phase was performed at the end of both interventions covering different static stretching exercises (e.g., standing forward fold, overhead triceps brachii stretch). Participants were allowed to continue with their usual physical activities during the 12-week intervention period.

### 2.5. Spatio-Temporal Gait Parameters

Stride length (m), gait velocity (m/s), the MTC (cm), and their respective coefficient of variation (CoV = 100 × standard deviation/mean [%]) were recorded during ST and DT walking using three inertial measurement units (XSENS MTW Awinda, Movella, Delft, The Netherlands). Gait data were recorded with a sampling frequency of 100 Hz and the sensors were placed proximally on each foot and the sternum. An algorithm developed by Hamacher et al. [55] was used to calculate the gait parameters.

### 2.6. Motor and Cognitive Dual-Task Costs

Both the walking and cognitive tasks were performed in a ST and DT condition. Bases on these data DTC were calculated as follows

(1) $$DTC=\frac{ST-DT}{ST}\times 100$$

(2) $$DTC=\frac{DT-ST}{DT}\times 100$$

In these formulas, ST and DT are the single- and dual-task performance for the walking and cognitive tasks. In case of a higher measurement value reflecting better performance, Equation (1) was used. Conversely, in case a lower measurement value reflecting better performance, Equation (2) was applied. Consequently, higher DTC are reflected by positive values indicative of a lower DT performance. On the contrary, negative values indicate lower DTC reflecting a higher DT compared to the ST performance.

### 2.7. Statistical Analysis

Data were analyzed using JASP Statistics (Version 0.19.3.0). Differences between groups in the anthropometric data were checked with independent t-tests. Since previous studies have shown the repeated measures analysis of covariance (ANCOVA) to be robust against moderate violation of normality [56] and variance heterogeneity [57], nonparametric tests were not used to check for differences.

Due to differences in the number of males and females between groups, sex was used as covariate. Three-way repeated measures analyses of covariance (ANCOVA) with the factors INTERVENTION (MMI, UMI), CONDITION (DT conditions [3 cognitive tasks × 2 levels of difficulty]), and TIME (pre, post) were conducted for both gait and cognitive outcomes. Further, to consider ST walking separately, a two-way repeated measures ANCOVA with the factors INTERVENTION (MMI, UMI) and TIME (pre, post) was performed for this data. To assess intervention effects on ST cognitive performance, a three-way repeated measures ANCOVA with the factors INTERVENTION (MMI, UMI), CONDITION (ST conditions [3 cognitive tasks × 2 levels of difficulty]), and TIME (pre, post) was conducted. If a violation of sphericity was detected, the Greenhouse-Geisser correction was used and Bonferroni-corrected post hoc tests were conducted in case of significant TIME × GROUP or main TIME effects.

To interpret the results and to clarify the practical/clinical relevance of interventional studies it is advisable to use effect sizes [58]. The effect size partial eta-squared ($\eta_{p}^{2}$) and Cohen’s d (d) were calculated and interpreted according to Lakens [58]: small ($\eta_{p}^{2}$ ≥ 0.01, d ≥ 0.2), medium ($\eta_{p}^{2}$ ≥ 0.06, d ≥ 0.5), and large ($\eta_{p}^{2}$ ≥ 0.14, d ≥ 0.8). Differences were interpreted as relevant if *p* ≤ 0.05 or $\eta_{p}^{2}$ ≥ 0.14. Post hoc comparisons were considered meaningful when the *p*-value was 0.05 or less or the effect size reached d ≥ 0.8. Data are presented as means ± standard deviations (SD), mean differences with 95% confidence intervals (CI) and 95% CI for d. Given the pilot nature of the present study, a post hoc sample size calculation was performed with G*Power (Version 3.1.9.7, Heinrich Heine University Düsseldorf, Düsseldorf, Germany) using the observed effect sizes to provide guidance on appropriate sample sizes for future randomized controlled trials on this topic (see Supplementary Tables S3–S5). Due to the explorative nature of the analysis in the present study, no correction for multiple testing was applied [59,60].

Additionally, to analyze the progression of the external load used during the resistance training (i.e., seated leg press and seated leg curls), the average load for each exercise was calculated on a monthly basis. a two-way repeated ANOVA was carried out with the factors INTERVENTION (MMI, UMI) and TIME (first, second, and third month). Furthermore, to analyze the progress of external load used during leg extension, Student’s *t*-test was calculated. The load progression served as an indirect marker of neuromuscular adaptations, which may particularly influence spatio-temporal gait parameters.

## 3. Results

In total, 19 glaucoma patients were recruited; however, due to dropouts only 15 participants completed the interventions (see Figure 1). A total of 8 participants (1 female, age: 69.3 ± 53 years, height: 171.8 ± 7.0 cm, body mass: 74.8 ± 13.5 kg) underwent MMI and seven participants (6 females, age: 70.1 ± 2.8 years, height: 166.4 ± 10.3 cm, body mass: 73.6 ± 24.0 kg) underwent UMI. The Freiburger Questionnaire on Physical Activity [46] showed that overall time of physical activity was on average 14 h for both the MMI and the UMI groups.

**Figure 2:**
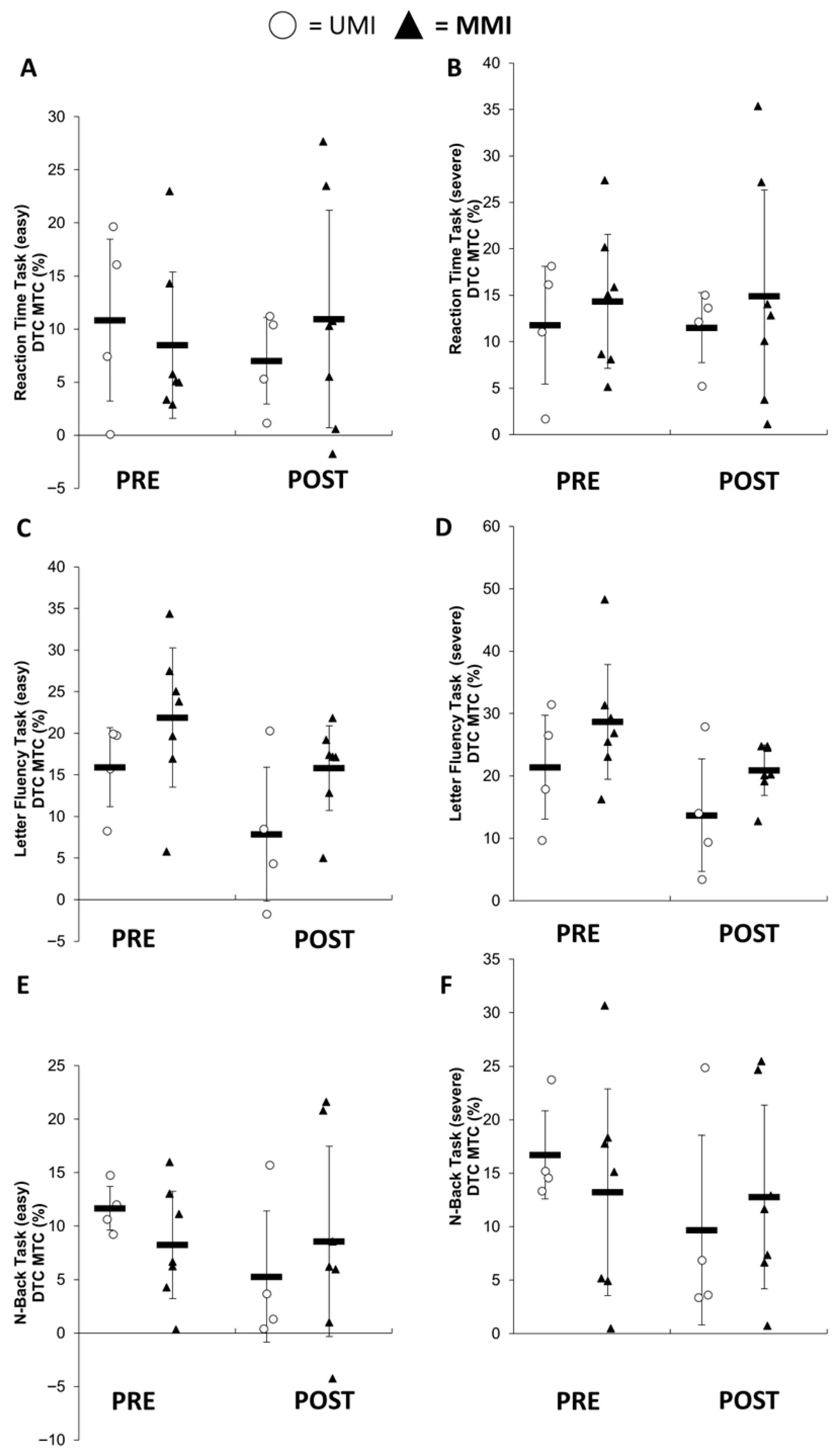
Means, standard deviations, and individual data for the dual-task costs minimal toe clearance (DTC MTC). Left-hand panels (**A**,**C**,**E**) represent the easy difficulty level, and right-hand panels (**B**,**D**,**F**) the severe difficulty level. DTC MTC was lower at post assessment for both groups: *p* = 0.188, $\eta_{p}^{2}$ = 0.205. DTC MTC was also lower for UMI compared to MMI at post assessment: *p* = 0.168, $\eta_{p}^{2}$ = 0.223. For details, see Supplementary Table S7.

**Figure 3:**
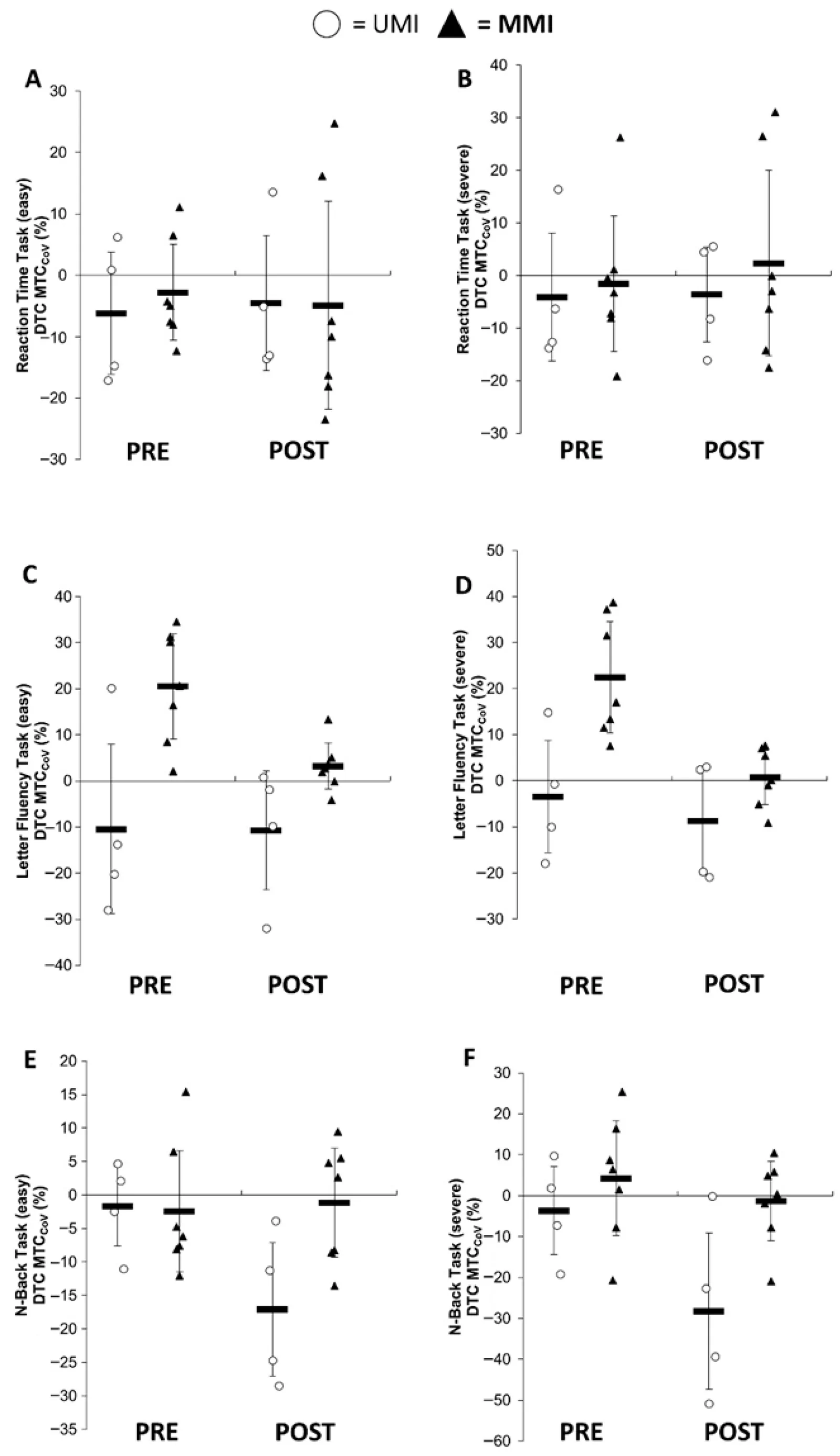
Means, standard deviations, and individual data for the dual-task costs minimal toe clearance coefficient of variance (DTC MTC_CoV_). Left-hand panels (**A**,**C**,**E**) represent the easy difficulty level, and right-hand panels (**B**,**D**,**F**) the severe difficulty level. DTC MTC_CoV_ was lower at post assessment for both groups: *p* = 0.002, $\eta_{p}^{2}$ = 0.713. DTC MTC_CoV_ was also lower for UMI compared to MMI at post assessment: *p* = 0.047, $\eta_{p}^{2}$ = 0.407. For details, see Supplementary Table S7.

Due to processing issues only 12 participants (7 MMI, 5 UMI) were included in the final DT gait analysis. Furthermore, due to technical issues during the cognitive tasks only 13 participants (8 MMI, 5 UMI) were included in the final analysis. Moreover, due to technical issues during ST walking trial at baseline measurement (day 1) only 11 participants (7 MMI, 4 UMI) were included into the final DTC analysis. For transparency, the number of analyzed cases for the respective parameter is shown in Supplementary Tables S6–S10 together with the main effects or interactions (see also Figure 2 and Figure 3). No difference in age (t(13) = 0.401, *p* = 0.695), height (t(13) = −1.203, *p* = 0.251), and weight (t(13) = −0.117, *p* = 0.909) was found between the groups. All participants had an attendance rate of the intervention of at least 80%. Post hoc sample size calculations based on the observed effect sizes of the present results are provided in Supplementary Tables S3–S5.

### 3.1. Dual-Task Analysis—Gait and Cognitive Parameters

The analysis of the DT walking trials including gait and cognitive performance showed neither TIME nor INTERVENTION × TIME effect.

### 3.2. Single-Task Analysis—Gait and Cognitive Parameters

Analysis of the ST data indicated a TIME effect for MTC_CoV_ (F(1,12) = 6.109, *p* = 0.029, $\eta_{p}^{2}$ = 0.337) and for cognitive performance (F(1,10) = 2.355, *p* = 0.156, $\eta_{p}^{2}$ = 0.191). However, post hoc analysis showed no difference between baseline and post measurement for MTC_CoV_ (*p* = 0.140, d = 0.217) and cognitive performance (*p* = 0.254, d = 0.100).

### 3.3. Dual-Task Costs Analysis—Gait Parameters

The analysis of the DTC showed TIME effects for DTC MTC (F(1,8) = 2.063, *p* = 0.188, $\eta_{p}^{2}$ = 0.205), DTC MTC_CoV_ (F(1,8) = 19.837, *p* = 0.002, $\eta_{p}^{2}$ = 0.713), and DTC stride length (F(1,8), *p* = 0.120, $\eta_{p}^{2}$ = 0.274).

Post hoc tests indicated decreased DTC MTC (*p* = 0.038, d = 0.439, mean difference = 3.532%, 95% CI = 0.240–6.824, 95% CI for d = 0.042–0.920) and, decreased DTC MTC_CoV_ (*p* = 0.034, d = 0.470, mean difference = 5.910%, 95% for CI = 0.586–11.235, 95% CI for d = 0.011–0.951) at post compared to baseline assessment. The post hoc tests revealed no TIME effect for DTC stride length (*p* = 0.566, d = 0.074).

The interaction of TIME × INTERVENTION was evident for DTC MTC (F(1,8) = 2.295, *p* = 0.168, $\eta_{p}^{2}$ = 0.223) and DTC MTC_CoV_ (F(1,8) = 5.484, *p* = 0.047, $\eta_{p}^{2}$ = 0.407). Post hoc tests revealed a lower DTC MTC for the UMI compared to the MMI at post (*p* = 0.326, d = 1.214, mean difference = −9.771%, 95% CI = −24.869–5.326, 95% CI for d = 3.367–0.939). Moreover, post hoc tests indicated a lower DTC MTC_CoV_ for the UMI at post compared to baseline (*p* = 0.073, d = 1.006, mean difference = 12.654%, 95% CI = −0.998–26.307%, 95% CI for d = 0.388–2.400).

Finally, post hoc tests revealed that the DTC MTC_CoV_ was lower at post for UMI than for MMI (*p* = 0.020, d = 1.590, mean difference = −19.994, 95% CI = −39.912–−3.077%, 95% CI for d = 3.132–0.048).

### 3.4. External Load Progression

The analysis of the external load progression showed no TIME × INTERVENTION interaction for seated leg press, seated leg curl or seated leg extensions. However, main effects of TIME were detected for seated leg press (F(1.146,14.900) = 12.683, *p* = 0.002, $\eta_{p}^{2}$ = 0.494) and seated leg curls (F(2,26) = 11.165, *p* < 0.001, $\eta_{p}^{2}$ = 0.462). Post hoc analysis showed a significant increase in the external load between the 1st and 2nd month for the seated leg press: (*p* = 0.007, d = 0.570, mean difference = 6.806 kg, 95% CI = 11.923–1.688%, 95% CI for d = 1.103–0.036). Furthermore, the post hoc analysis showed an increase in external load between the 1st and 2nd month for the leg curl (*p* < 0.001, d = 0.410, mean difference = 2.444 kg, 95% CI = 3.843–1.046%, 95% CI for d = 0.630–0.023).

## 4. Discussion

The present pilot-study compared the effect of a 12 weeks MMI (i.e., concurrent motor-cognitive DT training and resistance training) and UMI (i.e., resistance training only) on spatio-temporal gait parameters and cognitive performance in glaucoma patients during ST (i.e., walking without any additional task) and DT walking (i.e., walking while simultaneously performing a cognitive task). The main findings were as follows: both interventions had no significant effect on (i) gait performance or (ii) cognitive performance. However, it seems that independent of the intervention (iii) DTC MTC and (iv) DTC MTC_CoV_ improved over time, with (v) probably higher effects after UMI. Moreover, (vi) both interventions led to an increase in external load applied during the resistance training over the training period.

To the best of the authors’ knowledge, this is the first study comparing spatio-temporal gait parameters and cognitive performance after an MMI and UMI in glaucoma patients. With respect to gait and cognitive data, the present study showed no differences between baseline and post assessment. These results were not expected, given that positive effects of resistance training on gait performance and cognitive performance have already been reported [61,62]. In this context, Wollesen et al. [63] demonstrated that a progressive resistance training program combined with motor-cognitive DT exercises, such as fast walking with visual and balance tasks (60 min per session, 12 sessions, 12 weeks), led to improved gait performance, specifically increased step length, in healthy elderly individuals. Furthermore, Singh et al. [64] found that resistance training (75 min per session, 2–3 times per week, for 24 weeks) enhanced cognitive performance in patients with mild cognitive impairment. Additionally, Castano et al. [65] reported that performing resistance training simultaneously with a verbal fluency task (60 min, twice weekly, for 16 weeks) led to an increased blood plasma brain-derived neurotrophic factor level, a marker linked to neuroplasticity and neuroprotection [66,67], compared to conventional resistance training in healthy older adults. Based on the hypothesis that glaucoma may be associated with cognitive impairment [12], findings from an earlier cross-sectional study conducted by our research group revealed no significant differences in cognitive test performance between the present glaucoma patients and healthy control subjects [18]. This might be a possible explanation for the missing group effects. Furthermore, this might be supported by the mild stage of glaucoma progression in the present study (Figure 1).

However, the results of the present study indicate that both interventions improved the DTC MTC and DTC MTC_CoV_, with probably higher effects after UMI compared to the MMI, which might be related to neuromuscular adaptations indicated by the increase in external load used during resistance training. Although limited by the small sample size, data indicate that UMI might be more effective in reducing DTC compared to the MMI, which might be explained by the higher exercise volume [31] (Supplementary Table S2). The lack of superiority of the MMI compared to the UMI on the dependent outcomes might be due to the low dosage of the resistance training accompanying the Life Kinetik^®^ training [50]. Alternatively, Life Kinetik^®^ training [50] might not be the most suitable intervention to improve gait performance. For example, Hamacher [36] demonstrated that a six-month dance program led to a greater reduction in MTC_CoV_ compared to a combined exercise intervention including endurance, strength, and flexibility training in older adults. This effect may be attributed to the fact that dancing inherently integrates cognitive challenges into the motor task [68]. In contrast, the Life Kinetik^®^ program [50] introduces cognitive tasks as secondary distractors during motor tasks, i.e., which are not essential for completing the movement itself [42]. Therefore, interventions with cognitive engagement as a necessary component of motor execution may be more effective in enhancing gait performance than those in which cognitive tasks merely serve as distracting stimuli [42].

Interestingly, Freitag et al. have shown for healthy elderly controls that MMI and UMI failed to have an effect on gait performance during ST and DT walking (i.e., stride length, gait velocity, MTC, and their respective CoV) as well as cognitive performance [44]. Comparing these results with those of the present study indicates a higher adaptation and/or sensitivity of the MTC in glaucoma patients compared to healthy individuals in response to MMI and UMI. In this regard, our previous cross-sectional study comparing ST and DT walking performance of the same glaucoma patients and healthy controls revealed a reduced MTC, an increased MTC_CoV_, and an elevated DTC MTC during both ST and DT walking [18]. Therefore, the difference in MTC between glaucoma patients and healthy individuals, along with its changes in response to MMI and UMI, may serve as valuable prognostic indicators for assessing gait performance and potentially the risk of falls in people with glaucoma. However, because actual fall incidence was not assessed, it cannot be concluded that these biomechanical improvements translate into clinically meaningful reductions in falls. Future studies incorporating prospective fall monitoring are required to establish the clinical relevance of these findings.

The present study has some limitations. First, ST walking was consistently performed before DT walking, which might have promoted sequential effects. However, since the effects were systematic and consistent across participants, they are unlikely to have biased the group comparisons. Second, the absence of improvements in gait performance might be attributed to a lack of isolated training of the triceps surae muscle in both MMI and UMI. This muscle group plays a crucial role in plantar flexion and propulsive torque production during walking [69,70]. It is also particularly susceptible to age-related decline, contributing to reduced step length and increased reliance on hip and knee joints for power generation [71]. Additionally, the triceps surae muscle is important for maintaining gait stability following a stumble [72]. Therefore, incorporating more specific exercises for the plantar flexors may yield beneficial effects on gait performance [69,70,72]. Third, the assessors were not blinded to group allocation. Consequently, several aspects of the assessment process, including task administration, participant instruction, motivational influences, data processing, and data selection, may have been susceptible to observer-related bias. Fourth, due to overlap of the study period with the COVID-19 pandemic, the number of participants was relatively low [73] and, therefore, a more detailed analysis and interpretation of the results is complicated. Nevertheless, the results of this pilot study offer promising initial insights into the effects of both interventions on the considered outcomes. Fifth, the relatively small sample size and the unequal sex distribution between intervention groups. Although sex was included as a covariate in the ANCOVA models, the marked imbalance may have reduced the precision and stability of the estimated effects. Consequently, the findings should be interpreted with caution and considered exploratory. Nevertheless, ANCOVA is generally regarded as an appropriate analytical approach for continuous outcomes in randomized controlled trials because it can improve statistical precision and reduce bias by accounting for baseline variability [74]. Sixth, due to the pilot character and the small sample size of this pilot trial, an intention-to-treat analysis including imputation of missing data seemed not appropriate. Therefore, the robustness of the present findings to missing data was impossible to estimate.

Future research can build on these findings and should use the sample size estimates provided to validate and extend the observed outcomes. Notably, some of the sample size estimates presented in the Supplementary Tables S3–S5 are comparatively large, suggesting a considerable degree of uncertainty in the corresponding effect estimates. Therefore, these values should be interpreted with caution and may serve only as preliminary guidance for future research. In contrast, more moderate sample size estimates were observed for certain outcomes, such as minimum toe clearance (MTC), a well-established marker of gait stability. Further, these studies should also consider a different dosage of the resistance training and the Life Kinetik^®^ training [50] or other DT training programs (e.g., dancing [36]).

In conclusion, data of the present pilot study indicated that both MMI and UMI decreased DTC MTC and DTC MTC_CoV_ in glaucoma patients, with probably higher effects after UMI.

## Figures and Tables

**Table 1:** Ophthalmological characteristics of the participants.

		Multimodal Intervention (*n* = 10)	Unimodal Intervention (*n* = 9)	Difference
	Time	median/ range	median/ range	*p*-value
MD_right	Pre	−0.6/4.2	−0.0/14.0	1.00
	Post	−0.39/4.2	−0.4/14.2	0.86
MD_left	Pre	−1.2/20.4	−1.0/17.1	0.30
	Post	−1.7/20.5	−0.4/2.0	0.42
MD_binocular	Pre	0.5/4.0	0.4/8.9	0.95
	Post	0.3/4.5	0.7/7.6	0.42
		mean ± SD	mean ± SD	*p*-value
BCVA	Pre	−0.1 ± 0.2	−0.1 ± 0.1	0.71
	Post	−0.1 ± 0.1	−0.9 ± 0.1	0.49
pRNFL_right	Pre	79.4 ± 14.2	78.0 ± 11.8	0.84
	Post	N/A	N/A	
pRNFL_left	Pre	74.6 ± 16.3	77.6 ± 14.3	0.62
	Post	N/A	N/A	

BCVA = best corrected visual acuity [logMAR], pRNFL_right/left = peripapillary retinal nerve fiber layer (pRNFL) thickness [µm], MD_right/left = mean deviation of visual field [db], SD = standard deviation, N/A = not acquired.

## Data Availability

The datasets generated during the current study are not publicly available because of participant privacy and ethical restrictions but are available from the corresponding author upon reasonable request.

## Supplementary Materials

The following supporting information can be downloaded at: https://www.mdpi.com/article/10.3390/geriatrics11040095/s1, Table S1: Overview of the resistance exercises for the unimodal intervention and multimodal intervention throughout the intervention period. Letter in brackets indicate the major muscles involved in the exercise according to the Haff and Triplett (2016) [53]; Table S2: Multimodal intervention training schedule; Table S3: Sample size calculation for the different dual-task gait parameters based on the TIME × INTERVENTION effects, Table S4: Sample size calculation for the different single-task gait parameters based on the TIME × INTERVENTION effects; Table S5: Post-hoc power and sample size calculation for the different dual-task costs (DTC) based on the TIME × INTERVENTION effects; Table S6: Means ± standard deviations of the dual-task pre and post gait assessment as well as the outcomes of the ANCOVA; Table S7: Means ± standard deviations for the cognitive and motor dual task costs (DTC) of the pre and post measurement as well as the outcomes of the ANCOVA; Table S8: Means ± standard deviations of the single-task pre and post gait assessment as well as the outcomes of the ANCOVA; Table S9: Means ± standard deviations for the dual-task cognitive performance measures performed during the pre and post measurements as well as the outcomes of the ANCOVA. Table S10. Means ± standard deviations for the single-task cognitive performance measures performed during the pre and post measurements as well as the outcomes of the ANCOVA.

## Abbreviations

The following abbreviations are used in this manuscript:

ANCOVA	Analysis of covariance
CoV	Coefficient of variation
DT	Dual-task
DTC	Dual-task costs
Hz	Herz
MMI	Multimodal intervention
MTC	Minimum toe clearance
RPE	Rating of perceived exertion
ST	Single task
UMI	Unimodal intervention
95% CI	95% confidence interval
