# Supplementary material for: Effects of a Multimodal Exercise Intervention on Single- and Dual-Task Gait Performance in Older Patients with Open-Angle Glaucoma: A Pilot Study
Source: Geriatrics (Basel). 2026 Jul 30;11(4):95. doi: 10.3390/geriatrics11040095 (PMC13512113; doi:10.3390/geriatrics11040095)
Supplement: Supplementary file 1 [file geriatrics-11-00095-s001.zip › geriatrics-4335212-supplementary.pdf]

Table S1. Overview of the resistance exercises for the unimodal intervention and multimodal intervention throughout the intervention period. Letter in brackets indicate the major muscles involved in the exercise according to the Haff and Triplett (2016) [53]

<table>
<tr><th colspan="2">Week</th><th>Unimodal Intervention</th><th>Multimodal Intervention</th><th>Major Muscles Involved</th></tr>
<tr><td colspan="2">Weeks 1 – 4</td><td>1. Seated leg press (A)
2. Lever Pulldown (B)
3. Lever seated twist (C)
4. Lever chest press (D)
5. Cable curl (E)
6. Lever back extension (F)
7. Cable pushdown (G)
8. Cable upright row (H)
9. Seated leg curl (I)</td><td>1. Seated leg press (A)
2. Lever Pulldown (B)
3. Lever seated twist (C)
4. Lever chest press (D)
5. Cable curl (E)
6. Lever back extension (F)
7. Cable pushdown (G)
8. Cable upright row (H)
9. Seated leg curl (I)</td><td rowspan="3">A. Gluteus maximus, Hamstrings Quadriceps femoris
B. Latissimus dorsi, Teres major, Middle trapezius, Rhomboids, Posterior deltoids
C. Internal and external abdominal obliques
D. Pectoralis major, Anterior deltoids, Triceps brachii
E. Biceps brachii Brachialis, Brachioradialis
F. Erector spinae
G. Triceps brachii
H. Deltoids, Upper trapezius
I. Hamstrings
J. Quadriceps femoris
K. Deltoids
L. Latissimus dorsi, Triceps brachii</td></tr>
<tr><td rowspan="2">Weeks 5 – 8</td><td>Day 1</td><td>1. Seated leg press (A)
2. Cable straight back seated row (B)
3. Lever seated twist (C)
4. Lever chest press (D)
5. Cable curl (E)
6. Lever back extension (F)
7. Cable pushdown (G)
8. Cable upright row (H)
9. Lever leg extension (J)</td><td>1. Seated leg press (A)
2. Cable straight back seated row (B)
3. Lever seated twist (C)
4. Lever pec deck fly (D)
5. Cable upright row (H)
6. Cable curl (E)</td></tr>
<tr><td>Day 2</td><td>1. Seated leg press (A)
2. Lever pulldown (B)
3. Lever seated twist (C)
4. Lever pec deck fly (D)
5. Reverse cable curl (E)
6. Lever back extension (F)
7. Cable pushdown (G)
8. Dumbell seated lateral raise (K)
9. Seated leg curl (I)</td><td>1. Cable pushdown (G)
2. Lever pulldown (B)
3. Lever back extension (F)
4. Lever chest press (D)
5. Dumbbell seated lateral raise (K)
6. Seated leg curl (I)</td></tr>
</table>

**Weeks 9 – 12**	**Day 1**	1. Seated leg press (A)<br>2. Cable straight back seated row (B)<br>3. Lever chest press (D)<br>4. Lever seated twist (C)<br>5. Cable Curl (E)<br>6. Dumbbell seated front raise (K)<br>7. Seated leg curl (I)<br>8. Lever back extension (F)<br>9. Cable pushdown (G)	1. Seated leg press (A)<br>2. Dumbbell seated front raise (H)<br>3. Reverse cable curl (E)<br>4. Lever chest press (D)<br>5. Lever seated twist (C)	
	**Day 2**	1. Seated leg press (A)<br>2. Cable seated straight arm pulldown (L)<br>3. Lever seated twist (C)<br>4. Lever pec deck fly (D)<br>5. Reverse cable curl (E)<br>6. Seated triceps press (G)<br>7. Dumbbell seated lateral raise (K)<br>8. Lever leg extension (J)<br>9. Lever back extension (F)	1. Seated leg curl (I)<br>2. Lever pec deck fly (D)<br>3. Lever back extension (F)<br>4. Seated triceps press (G)<br>5. Cable seated straight arm pulldown (L)	

Table S2. Multimodal intervention training schedule

Training components	Months		
	1st Month	2nd Month	3rd Month
Life Kinetik®	10 min	20 min	30 min
Resistance Training	40 min	30 min	20 min
Cool Down	10 min	10 min	10 min

Table S3. Sample size calculation for the different dual-task gait parameters based on the TIME x INTERVENTION effects

Outcome Measures	N (MMI-UMI)	p	η_p^2	Required Total Sample Size
MTC (cm)	12	0.421	0.073	44
MTC_{CoV} (%)	12	0.754	0.012	296
Stride Length (m)	12	0.442	0.067	484
Stride $Length_{CoV}$ (%)	12	0.448	0.065	50
Gait Velocity (m/s)	12	0.889	0.002	1624
Gait $Velocity_{CoV}$ (%)	12	0.372	0.089	36
Cognitive Performance	13	0.680	0.018	180

Sample size calculation (test family = F-test, statistical test = repeated measures ANOVA, type of power analysis = post hoc, $\alpha = 0.05$, power ($1 - \beta$ error probability) threshold = 0.95, number of groups = 2, number of measurements = 2, correction among repeated measures = 0.5, $\epsilon = 1$) based on the found TIME x INTERVENTION effect sizes for the different outcomes.

Table S4. Sample size calculation for the different single-task gait parameters based on the TIME x INTERVENTION effects

Outcome Measures	N (MMI-UMI)	p	η_p^2	Required Total Sample Size
MTC (cm)	15	0.891	0.002	230
MTC_{CoV} (%)	15	0.529	0.034	16
Stride Length (m)	15	0.700	0.013	38
Stride $Length_{CoV}$ (%)	15	0.237	0.114	6
Gait Velocity (m/s)	15	0.847	0.003	154
Gait $Velocity_{CoV}$ (%)	15	0.338	0.077	8
Cognitive Performance	15	0.408	0.070	10

Sample size calculation (test family = F-test, statistical test = repeated measures ANOVA, type of power analysis = post hoc, α = 0.05, power (1 – β error probability) threshold = 0.95, number of groups = 2, number of measurements = 2, correction among repeated measures = 0.5, ϵ = 1) based on the found TIME x INTERVENTION effect sizes for the different outcomes.

Table S5. Post-hoc power and sample size calculation for the different dual-task costs (DTC) based on the TIME x INTERVENTION effects

Outcome Measures	N (MMI-UMI)	p	η_p^2	Required Total Sample Size
DTC MTC (cm)	11	0.168	0.223	14
DTC MTC_{CoV} (%)	11	0.047	0.407	8
DTC Stride Length (m)	11	0.531	0.051	64
DTC Stride $Length_{CoV}$ (%)	11	0.450	0.070	46
DTC Gait Velocity (m/s)	11	0.793	0.009	360
DTC Gait $Velocity_{CoV}$ (%)	11	0.753	0.013	250
DTC Cognitive Performance	12	0.507	0.045	72

Sample size calculation (test family = F-test, statistical test = repeated measures ANOVA, type of power analysis = post hoc, $\alpha = 0.05$, power ($1 - \beta$ error probability) threshold = 0.95, number of groups = 2, number of measurements = 2, correction among repeated measures = 0.5, $\epsilon = 1$) based on the found TIME x INTERVENTION x CONDITION effect sizes for the different outcomes.

Table S6. Means ± standard deviations of the dual-task pre and post gait assessment as well as the outcomes of the ANCOVA

Time	Condition/ Intervention	Letter Fluency Task (easy)	Letter Fluency Task (severe)	Reaction Time Task (easy)	Reaction Time Task (severe)	N-Back Task (easy)	N-Back Task (severe)	Repeated Measures ANCOVA	
								Time	***Time x Intervention***
	**MTC (cm)**								
PRE	MMI N = 7	1.798 ± 0.407	1.633 ± 0.355	2.012 ± 0.272	1.887 ± 0.292	2.066 ± 0.314	1.948 ± 0.330	$F_{1,9} = 0.254$ p = 0.626 $\eta_p^2 = 0.027$	$F_{1,9} = 0.712$ p = 0.421 $\eta_p^2 = 0.073$
	UMI N = 5	1.987 ± 0.500	1.864 ± 0.466	2.183 ± 0.499	2.127 ± 0.497	2.180 ± 0.380	2.017 ± 0.402		
POST	MMI N = 7	1.774 ± 0.375	1.672 ± 0.383	1.984 ± 0.460	1.900 ± 0.487	1.993 ± 0.448	1.902 ± 0.427		
	UMI N = 5	2.196 ± 0.533	2.033 ± 0.560	2.272 ± 0.353	2.156 ± 0.370	2.201 ± 0.484	2.081 ± 0.540		
	**MTC_{CoV} (%)**								
PRE	MMI N = 7	41.228 ± 11.177	42.764 ± 12.768	32.565 ± 7.268	33.106 ± 6.847	32.085 ± 6.577	34.961 ± 9.103	$F_{1,9} = 1.103$ p = 0.756 $\eta_p^2 = 0.011$	$F_{1,9} = 0.104$ p = 0.754 $\eta_p^2 = 0.011$
	UMI N = 5	33.531 ± 7.004	35.477 ± 7.301	33.360 ± 2.834	35.676 ± 6.231	32.024 ± 3.216	32.556 ± 5.312		
POST	MMI N = 7	38.773 ± 8.996	38.116 ±10.639	32.930 ± 5.510	35.629 ± 6.624	35.014 ± 6.766	35.453 ± 9.627		
	UMI N = 5	33.893 ± 11.718	35.941 ± 14.507	31.815 ± 7.618	33.113 ±10.018	31.840 ± 8.160	31.638 ± 11.488		
	**Stride Length (m)**								
PRE	MMI N = 7	1.204 ± 0.164	1.192 ± 0.159	1.274 ± 0.149	1.274 ± 0.145	1.263 ± 0.178	1.253 ± 0.164	$F_{1,9} = 1.312$ p = 0.282 $\eta_p^2 = 0.127$	$F_{1,9} = 0.647$ p = 0.442 $\eta_p^2 = 0.067$
	UMI N = 5	1.316 ± 0.073	1.312 ± 0.064	1.346 ± 0.087	1.361 ± 0.072	1.361 ± 0.072	1.332 ± 0.062		
POST	MMI N =7	1.212 ± 0.152	1.201 ± 0.152	1.273 ± 0.141	1.276 ± 0.138	1.286 ± 0.146	1.275 ± 0.141		

	UMI<br>N = 5	1.334<br>± 0.078	1.330<br>± 0.067	1.375<br>± 0.069	1.381<br>± 0.062	1.372<br>± 0.075	1.345<br>± 0.063		
	**Stride Length$_{CoV}$ (%)**								
PRE	MMI<br>N = 7	13.517<br>± 1.983	13.613<br>± 2.346	13.679<br>± 2.188	13.522<br>± 2.357	13.142<br>± 1.595	12.900<br>± 1.613	$F_{1,9}$ = 0.000<br>p = 0.993<br>η_p^2 = 0.000	$F_{1,9}$ = 0.630<br>p = 0.448<br>η_p^2 = 0.065
	UMI<br>N = 5	12.898<br>± 2.918	13.372<br>± 2.869	13.895<br>± 2.177	14.138<br>± 1.977	13.812<br>± 1.810	13.808<br>± 2.128		
POST	MMI<br>N = 7	13.844<br>± 2.290	12.940<br>± 2.027	12.438<br>± 2.068	12.507<br>± 2.156	12.953<br>± 2.203	12.848<br>± 1.854		
	UMI<br>N = 5	13.977<br>± 3.196	14.200<br>± 3.285	14.379<br>± 2.066	14.656<br>± 2.596	13.878<br>± 1.400	13.706<br>± 1.485		
	**Gait Velocity (m/s)**								
PRE	MMI<br>N = 7	1.007<br>± 0.183	0.966<br>± 0.204	1.134<br>± 0.140	1.135<br>± 0.133	1.127<br>± 0.173	1.114<br>± 0.162	$F_{1,9}$ = 0.998<br>p = 0.344<br>η_p^2 = 0.100	$F_{1,9}$ = 0.020<br>p = 0.889<br>η_p^2 = 0.002
	UMI<br>N = 5	1.121<br>± 0.081	1.228<br>± 0.092	1.299<br>± 0.125	1.332<br>± 0.133	1.334<br>± 0.156	1.266<br>± 0.105		
POST	MMI<br>N = 7	1.036<br>± 0.155	1.026<br>± 0.161	1.144<br>± 0.136	1.140<br>± 0.139	1.158<br>± 0.147	1.146<br>± 0.155		
	UMI<br>N = 5	1.259<br>± 0.117	1.234<br>± 0.122	1.327<br>± 0.141	1.345<br>± 0.143	1.343<br>± 0.146	1.279<br>± 0.106		
	**Gait Velocity$_{CoV}$ (%)**								
PRE	MMI<br>N = 7	16.386<br>± 3.027	15.566<br>± 2.456	15.566<br>± 2.943	15.346<br>± 2.634	15.280<br>±2.323	15.069<br>± 2.312	$F_{1,9}$ = 0.012<br>p = 0.916<br>η_p^2 = 0.001	$F_{1,9}$ = 0.881<br>p = 0.372<br>η_p^2 = 0.089
	UMI<br>N = 5	14.736<br>± 3.417	15.817<br>± 3.514	15.329<br>± 2.310	15.332<br>± 2.147	14.962<br>± 2.077	15.371<br>± 2.221		
POST	MMI<br>N = 7	16.191<br>± 3.232	15.674<br>± 2.995	14.389<br>± 2.683	14.387<br>± 2.525	14.862<br>± 2.481	14.891<br>± 2.337		
	UMI<br>N = 5	15.689<br>± 3.220	16.346<br>± 3.569	16.077<br>± 2.960	15.977<br>± 3.052	15.619<br>± 2.463	15.765<br>± 2.658		

Table S7. Means ± standard deviations for the cognitive and motor dual task costs (DTC) of the pre and post measurement as well as the outcomes of the ANCOVA

Time	Condition/ Group	Letter Fluency Task (easy)	Letter Fluency Task (severe)	Reaction Time Task (easy)	Reaction Time Task (severe)	N-Back Task (easy)	N-Back Task (severe)	DTC Repeated Measures ANCOVA	
								Time	***Time x Intervention***
	**DTC MTC (%)**								
PRE	MMI N = 7	21.893 ± 9.034	28.671 ± 9.915	8.485 ± 7.447	14.351 ± 7.779	8.246 ± 5.423	13.220 ± 10.421	$F_{1,8}$ = 2.063, p = 0.189, η_p^2 = 0.205	$F_{1,9}$ = 0.295, p = 0.168, η_p^2 = 0.223
	UMI N = 4	15.929 ± 5.475	21.391 ± 9.605	10.822 ± 8.799	11.780 ± 7.337	11.668 ± 2.364	16.717 ± 4.746		
POST	MMI N = 7	15.811 ± 5.479	20.917 ± 4.349	10.945 ± 11.069	14.926 ± 12.319	8.564 ± 9.608	12.781 ± 9.271		
	UMI N = 4	7.856 ± 9.299	13.705 ± 10.430	7.019 ± 4.713	11.513 ± 4.346	5.283 ± 7.086	9.694 ± 10.238		
	**DTC MTC_{CoV} (%)**								
PRE	MMI N = 7	20.522 ± 12.319	22.438 ± 13.032	-2.807 ± 8.437	-1.521 ± 13.939	-2.397 ± 9.732	4.286 ± 15.222	$F_{1,8}$ = 19.386, p = 0.002, η_p^2 = 0.713	$F_{1,8}$ = 5,484 p = 0.047, η_p^2 = 0.407
	UMI N = 4	-10.467 ± 21.211	-3.436 ± 14.097	-6.187 ± 11.496	-4.060 ± 14.016	-1.671 ± 6.899	-3.706 ±12.424		
POST	MMI N = 7	3.218 ± 5.399	0.735 ± 6.381	-4.896 ± 18.294	2.371 ± 19.083	-1.130 ± 8.804	-1.301 ± 10.465		
	UMI N = 4	-10.742 ± 14.860	-8.759 ± 13.312	-4.518 ± 12.691	-3.547 ± 10.415	-17.074 ± 11.484	-28.244 ± 22.003		
**DTC Stride Length (%)**									
PRE	MMI N = 7	8.897 ± 4.570	9.778 ± 4.570	2.617 ± 2.587	2.530 ± 2.897	3.030 ± 2.726	3.613 ± 4.305	$F_{1,8}$ = 3.019, p = 0.120, η_p^2 = 0.274	$F_{1,8}$ = 0.428, p = 0.531, η_p^2 = 0.051
	UMI N = 4	4.924 ± 1.172	5.351 ± 1.531	2.926 ± 2.492	1.293 ± 1.062	2.161 ± 2.536	3.897 ± 3.981		
POST	MMI N = 7	6.484 ± 5.450	7.331 ± 4.929	3.971 ± 3.370	3.737 ± 3.447	2.529 ± 2.902	3.257 ± 4.148		

	UMI<br>N = 4	4.756<br>± 3.601	4.872<br>± 3.352	2.333<br>± 1.586	1.648<br>± 1.014	2.118<br>± 1.238	3.677<br>± 1.966		
**DTC Stride Length$_{CoV}$ (%)**									
PRE	MMI<br>N = 7	-12.576<br>± 14.280	-11.735<br>± 10.325	-10.406<br>± 11.226	-12.116<br>± 14.234	-7.819<br>± 10.559	-9.736<br>± 9.456	$F_{1,8}$ = 1.151,<br>p = 0.315,<br>η_p^2 = 0.126	$F_{1,8}$ = 0.060,<br>p = 0.460,<br>η_p^2 = 0.070
	UMI<br>N = 4	-24.903<br>± 21.861	-24.206<br>± 19.730	-10.595<br>± 6.869	-10.593<br>± 6.770	-10.881<br>± 10.170	-12.183<br>± 16.939		
POST	MMI<br>N = 7	-4.359<br>± 10.335	-11.560<br>± 10.653	-12.478<br>± 13.006	-12.434<br>± 18.102	-8.830<br>± 8.689	-9.513<br>± 12.914		
	UMI<br>N = 4	-20.565<br>± 21.231	-18.960<br>± 18.579	-8.136<br>± 7.710	-6.964<br>± 4.821	-15.780<br>±13.927	-19.095<br>± 18.843		
**DTC Gait Velocity (%)**									
PRE	MMI<br>N = 7	15.747<br>± 8.020	19.121<br>± 11.423	3.687<br>± 4.344	3.524<br>± 5.583	3.080<br>± 4.482	3.936<br>± 7.073	$F_{1,8}$ = 0.235,<br>p = 0.641,<br>η_p^2 = 0.029	$F_{1,8}$ = 0.074,<br>p = 0.793,<br>η_p^2 = 0.001
	UMI<br>N = 4	8.484<br>± 4.548	9.800<br>± 6.136	4.215<br>± 4.618	2.469<br>± 3.958	2.971<br>± 5.330	6.745<br>± 8.693		
POST	MMI<br>N = 7	10.641<br>± 6.528	11.669<br>± 5.779	5.851<br>± 5.864	6.177<br>± 6.671	2.606<br>± 5.304	3.547<br>± 7.597		
	UMI<br>N = 4	7.400<br>5.610	9.112<br>± 7.178	2.558<br>± 1.881	1.392<br>± 0.912	3.165<br>± 4.339	6.690<br>± 5.359		
**DTC Gait Velocity$_{CoV}$ (%)**									
PRE	MMI<br>N = 7	-7.954<br>± 9.822	-12.824<br>± 3.215	-12.660<br>± 9.191	-13.966<br>± 9.781	-9.920<br>± 7.838	-11.493<br>± 8.762	$F_{1,8}$ = 0.579,<br>p = 0.469,<br>η_p^2 = 0.067	$F_{1,8}$ = 0.106,<br>p = 0.753,<br>η_p^2 = 0.013
	UMI<br>N = 4	-17.000<br>± 18.734	-11.678<br>± 12.868	-10.615<br>± 3.898	-10.955<br>± 3.473	-12.673<br>± 9.306	-9.838<br>± 11.600		
POST	MMI<br>N = 7	-4.502<br>± 10.319	-7.928<br>± 10.529	-15.527<br>± 12.776	-15.891<br>± 18.866	-12.998<br>± 10.007	-12.548<br>± 10.740		
	UMI<br>N = 4	-13.350<br>± 14.365	-11.012<br>± 9.584	-8.630<br>± 6.414	-7.970<br>± 4.231	-14.305<br>± 13.935	-13.611<br>± 20.592		
**DTC Cognitive Performance (%)**									

PRE	MMI N = 8	8.887 ± 16.799	7.854 ± 24.454	-0.965 ± 9.045	2.778 ± 5.966	-4.976 ± 12.740	2.698 ± 11.814	$F_{1,10}$ = 0.121, p = 0.735, η_p^2 = 0.012	$F_{1,10}$ = 0.089, p = 0.771, η_p^2 = 0.009
	UMI N = 5	0.572 ± 12.984	8.207 ± 12.820	3.333 ±2.977	5.694 ± 6.304	2.167 ± 6.937	15.736 ± 14.485		
POST	MMI N = 8	0.016 ± 19.460	3.753 ± 17.317	-4.737 ± 9.258	2.478 ± 8.639	0.833 ± 1.543	-3.880 ± 12.039		
	UMI N = 5	-4.090 ± 21.676	-2.201 ± 11.589	-3.344 ± 7.677	5.124 ± 7.531	-0.670 ± 1.542	--28.374 ± 74.113		

Table S8. Means ± standard deviations of the single-task pre and post gait assessment as well as the outcomes of the ANCOVA

Time	Gait parameter/ Intervention	**MTC (cm)**	**MTC_{CoV} (%)**	**Stride length (m)**	**Stride $length_{CoV}$ (%)**	**Gait velocity (m/s)**	**Gait $velocity_{CoV}$ (%)**
PRE	MMI N = 8	2.293 ± 0.357	35.336 ± 7.805	1.298 ± 0.159	14.947 ± 2.378	1.163 ± 0.146	17.488 ± 2.561
	UMI N = 7	2.290 ± 0.581	32.379 ± 7.167	1.355 ± 0.127	15.275 ± 2.942	1.327 ± 0.197	16.945 ± 3.174
POST	MMI N = 8	2.244 ±0.368	35.616 ± 7.547	1.312 ± 0.140	14.413 ± 2.305	1.188 ± 0.111	17.310 ± 3.442
	UMI N = 7	2.379 ± 0.511	35.549 ± 7.465	1.384 ± 0.081	16.028 ± 2.392	1.366 ± 0.135	17.654 ± 2.892
Repeated Measures ANCOVA	TIME	$F_{1,12} = 0.218$ $p = 0.649$ $\eta_p^2 = 0.018$	$F_{1,12} = 6.109$ $p = 0.029$ $\eta_p^2 = 0.337$	$F_{1,12} = 0.497$ $p = 0.494$ $\eta_p^2 = 0.040$	$F_{1,12} = 0.593$ $p = 0.456$ $\eta_p^2 = 0.047$	$F_{1,12} = 0.061$ $p = 0.808$ $\eta_p^2 = 0.005$	$F_{1,12} = 0.542$ $p = 0.476$ $\eta_p^2 = 0.004$
	TIME x INTERVENTION	$F_{1,12} = 0.020$ $p = 0.891$ $\eta_p^2 = 0.002$	$F_{1,12} = 0.421$ $p = 0.529$ $\eta_p^2 = 0.034$	$F_{1,12} = 0.156$ $p = 0.700$ $\eta_p^2 = 0.013$	$F_{1,12} = 1.546$ $p = 0.237$ $\eta_p^2 = 0.114$	$F_{1,12} = 0.039$ $p = 0.847$ $\eta_p^2 = 0.003$	$F_{1,12} = 0.998$ $p = 0.338$ $\eta_p^2 = 0.077$

Table S9. Means ± standard deviations for the dual-task cognitive performance measures performed during the pre and post measurements as well as the outcomes of the ANCOVA

Time	Condition/ Intervention	Dual-Task					
		Letter Fluency Task (n) (easy)	Letter Fluency Task (n) (severe)	Reaction Time Task (s) (easy)	Reaction Time Task (s) (severe)	N-Back Task (n) (easy)	N-Back Task (n) (severe)
PRE	MMI N = 8	38.125 ± 13.250	31.250 ± 14.665	0.979 ± 0.154	0.937 ± 0.126	29.875 ± 0.353	24.875 ± 4.016
	UMI N = 5	35.200 ± 10.826	27.400 ± 3.912	0.992 ± 0.079	0.938 ± 0.075	28.800 ± 2.683	20.400 ± 5.030
POST	MMI N = 8	41.125 ± 13.021	33.500 ± 13.082	0.861 ± 0.158	0.849 ± 0.156	29.750 ± 0.463	24.750 ± 3.732
	UMI N = 5	40.400 ± 9.839	33.600 ± 7.635	0.885 ± 0.252	0.885 ± 0.252	30.000 ± 0.000	25.000 ± 2.000

Repeated Measures ANCOVA	
Time	***Time x Intervention***
$F_{1,10} = 0.005$, $p = 0.950$, $\eta_p^2 = 0.000$	$F_{1,10} = 0.180$, $p = 0.680$, $\eta_p^2 = 0.018$

Table S10. Means ± standard deviations for the single-task cognitive performance measures performed during the pre and post measurements as well as the outcomes of the ANCOVA

Time	Condition/ Intervention	Single-Task					
		Letter Fluency Task (n) (easy)	Letter Fluency Task (n) (severe)	Reaction Time Task (s) (easy)	Reaction Time Task (s) (severe)	N-Back Task (n) (easy)	N-Back Task (n) (severe)
PRE	MMI N = 8	41.000 ± 8.992	33.250 ± 12.233	0.984 ± 0.134	0.912 ± 0.138	27.750 ± 2.765	25.625 ± 3.739
	UMI N = 5	35.800 ± 12.194	30.800 ± 8.672	0.957 ± 0.049	0.881 ± 0.027	29.400 ± 0.894	24.000 ± 2.915
POST	MMI N = 8	43.125 ± 15.887	35.875 ± 14.055	0.898 ± 0.157	0.823 ± 0.131	30.000 ± 0.000	24.250 ± 5.175
	UMI N = 5	39.600 ± 10.550	32.600 ± 4.393	0.904 ± 0.236	0.808 ± 0.195	29.800 ± 0.447	22.800 ± 7.294

Repeated Measures ANCOVA	
Time	***Time x Intervention***
$F_{1,10} = 2.355$, $p = 0.156$, $\eta_p^2 = 0.191$	$F_{1,10} = 0.746$, $p = 0.408$, $\eta_p^2 = 0.070$
